# Selecting and Testing Environmental Enrichment in Lemurs

**DOI:** 10.3389/fpsyg.2019.02119

**Published:** 2019-09-13

**Authors:** Eduardo J. Fernandez, William Timberlake

**Affiliations:** ^1^School of Behavior Analysis, Florida Institute of Technology, Melbourne, FL, United States; ^2^Department of Psychological and Brain Sciences, Center for the Integrative Study of Animal Behavior, Indiana University, Bloomington, IN, United States

**Keywords:** animal welfare, enrichment, lemur, paired-choice, preference assessment, zoo

## Abstract

Environmental enrichment has become a standard tool for improving the welfare of animals in zoos. Two critical steps in the manipulation of environmental enrichment are (1) selection of objects/procedures and (2) evaluation of their effects. In this study, we examined the selection and evaluation of feeding enrichment for four species of lemur. Experiment 1 used a paired-choice preference assessment to divide eight food items into high- and low-preferred categories. Experiment 2 separately assessed the effects of high- versus low-preferred items (placed in bamboo dispensers) on the behavior of two of the species in the preference assessment. Both high- and low-preferred items increased general activity and overall enclosure use, with high-preferred items having a greater effect than low-preferred items on most measures. The results suggest that preference assessments can serve as useful tools in selecting potential enrichment and that enrichment testing is important in evaluating the significance of these preferences.

Over the past several decades, the use of environmental enrichment to promote the health and well-being of animals in zoos and other captive settings has increased (Markowitz and Aday, 1998; Shepherdson, 1998; Mellen and MacPhee, 2001). Advantages of environmental enrichment include (1) reducing stereotyped and aberrant behaviors, (2) improving the general health and increasing the longevity of captive species, and (3) promoting more naturalistic behaviors (Markowitz, 1978; Carlstead, 1996). In addition, the display of naturalistic behaviors in zoo animals (the goal of enrichment) has been correlated with increased visitor attention and perceived likability of the animal/exhibit by the visitor (Finlay et al., 1988; Altman, 1998).

Examples of environmental enrichment include the presentation of food items to felids (Shepherdson et al., 1993; Lyons et al., 1997) and bears (Law et al., 1990; Carlstead et al., 1991; Forthman et al., 1992). Artificial foraging objects have also been presented, including acoustic “prey” for African leopards (Markowitz et al., 1995) and manipulable objects for bears (Altman, 1999). Modifying the captive environment has been tried as well, including presenting a species with a new exhibit or rotating a species through different exhibits (Chang et al., 1999; Little and Sommer, 2002; Lukas et al., 2003).

Introducing environmental enrichment for captive animals has two critical aspects: enrichment selection (choosing potential enrichment) and enrichment evaluation (measuring the effects of the enrichment). In the case of enrichment selection, choices are often made based on keeper/caretaker opinion and anecdotal reports of past successes. Only a few researchers have suggested a systematic basis for the selection of potential enrichment items (Mellen and MacPhee, 2001; Fernandez et al., 2004; Alligood et al., 2017). In the case of enrichment evaluation, researchers have emphasized the systematic assessment of enrichment in relation to the psychological well-being and behavior of captive animals (Crockett, 1998; Morgan et al., 1998; Shepherdson, 1998), but this remains a relatively newer component of animal welfare assessment, with a growing need for data-driven enrichment evaluation.

The present study investigated systematic procedures for selecting and evaluating feeding enrichment manipulations in several species of captive lemurs: ring-tailed lemurs (*Lemur catta*), red ruffed lemurs (*Varecia rubra*), collared brown lemurs (*Eulemur collaris*), and blue-eyed black lemurs (*E. flavifrons*). Lemurs are exclusively found in the island of Madagascar, off the southeastern coast of Africa. They are highly social primates, evading predation by foraging in groups, and are primarily nocturnal and arboreal. (Jolly, 1966; van Schaik and Kappeler, 1993; Scheumann et al., 2007). While most lemur species are nocturnal and arboreal, ring-tailed lemurs are known to forage diurnally and terrestrially (Gould and Sauther, 2007; see General Discussion). As such, we were additionally interested in differences that might exist in the latter enrichment evaluation for the ring-tailed lemurs.

To select our enrichment manipulation, we systematically assessed preferences for various food items (Young and Chaplin, 1945; Young and Kappauf, 1962; Thompson and Grant, 1971). Applied researchers have used similar assessments to determine human preferences for potential reinforcers (Pace et al., 1985; DeLeon and Iwata, 1996; Roscoe et al., 1999). These assessments include single-, paired-, and multiple-stimulus methods (see Fisher and Mazur, 1997 for a review). We chose the paired-choice procedure because it can rapidly rank order stimulus preferences and can readily be administered to non-human animals. In this method, items are repeatedly and concurrently presented in pairs to an individual who selects one of them. After all possible combinations are presented, the researchers rank the items based on the percentage of times an individual selected each item (Fisher et al., 1992).

The paired-choice procedure has been applied previously in zoo settings. For example, Fernandez et al. (2004) used the procedure to determine food preferences of five cotton-top tamarins. Similar studies documented browse preferences for five colobus monkeys (Tovar et al., 2005), preferences among three species of bamboo in a pair of giant pandas (Tarou et al., 2005), preferences for training or enrichment in wolves (Dorey et al., 2015), object and interaction preferences and enrichment efficacy in Galapagos tortoises (Mehrkam and Dorey, 2014), scent preferences in giraffes (Fay and Miller, 2015), preferences for potential enrichment items with several species of zoo-housed animals (Mehrkam and Dorey, 2015), and with domestic cats and dogs in other applied animal settings (Vicars et al., 2014; Vitale Shreve et al., 2017). However, apart from Mehrkam and Dorey (2014), these studies did not attempt to evaluate the relationship of enrichment selection through preference assessments to the success of subsequent enrichment introductions, and none of the above studies directly compared preference order to their resultant enrichment effectiveness.

In this study, we assessed paired-choice preferences for food items, ranking the items as high-preferred (HP) or low-preferred (LP). We then evaluated the enrichment effects of these items on the lemurs’ foraging behavior and general activity. Experiment 1 assessed preferences for paired selection and consumption of eight food items in four species of lemurs. Experiment 2 placed high- and low-preferred items (based on Experiment 1) in bamboo dispensers and tested the effect of presenting filled versus empty dispensers to a mixed group of ring-tailed and collared lemurs (*Lemur catta* and *Eulemur collaris*, respectively) in their outdoor exhibit.

We hypothesized that enrichment effects should be greater for high- vs. low-preferred conditions, and higher for food vs. non-food [Baseline (BL)] conditions. We expected this greater enrichment effect to be observed *via* (1) increased foraging and general activity, and (2) greater overall enclosure use.

## Experiment 1: Enrichment Selection

### Method

#### Subjects and Enclosures

Nineteen adult lemurs (age range: 4–10 years) across four species were included in the study: seven ring-tailed (two male, five female; *Lemur catta*), seven red ruffed (five male, two female; *Varecia rubra*), three collared (one male, two female; *Eulemur collaris*), and two blue-eyed black lemurs (one male, one female; *E. flavifrons*). All lemurs were captive-born and housed at the Indianapolis Zoo. All lemurs were approved for use in this study by the Indiana University – Bloomington Institutional Animal Care and Use Committee (IACUC; Study # 04-116), as well as through the Indianapolis Zoo’s internal research review process.

The seven red ruffed and two blue-eyed black lemurs resided in a 185 m^2^ enclosed outdoor island exhibit during the day. Three of the ring-tailed and all three collared lemurs resided in a 97 m^2^ outdoor island exhibit during the day. The ring-tailed/collared lemur exhibit contained a 2 m × 1.5 m × 1 m artificial rock that was hollow in front, allowing the lemurs to move under the structure yet remain visible to the public. The red ruffed/blue-eyed black lemur exhibit contained several 0.5 m × 1 m × 0.5 m hollow logs. Both exhibits contained trees, branches, or similar fixed hanging structures for the lemurs to move across while being viewed by the public.

At night or when the temperature remained below 21°C, lemurs were separated by species and placed in holding enclosures. The red ruffed and blue-eyed black lemur holding enclosure was approximately 155 m^2^. The ring-tailed and collared lemur holding enclosure was approximately 123 m^2^. The final four ring-tailed lemurs were a breeding group and were maintained in a similar holding enclosure throughout the day. All trials were run in the holding/night enclosures.

#### Materials

Eight food items were used during the preference assessment: zucchini, cauliflower, red pepper, green beans, corn, yams, eggplant, and squash. These food items were selected because they were part of the lemurs’ standard diet and were desired by the management/staff to be used in enrichment procedures. Each food item was cut into 2–3 cm^2^ × 2–3 cm^2^. During a trial, two items were placed on a 50 cm × 25 cm tray approximately 35–40 cm apart. Data sheets listing order and choices for each trial were used to record the food selections.

#### Data Collection and Procedures

The paired-choice preference assessment in this study used methods like those of Fisher et al. (1992) for presenting stimuli in concurrent pairs. All lemurs were initially allowed to approach and sample each of the eight food items as a keeper presented each one individually on the tray. A list of pairs for all eight items was generated, presenting each food item on the left against all seven other food items and again for the right side; therefore, each food item was equally presented with each other food item on both the right and left sides (14 presentations for each food item, and a total of 56 food presentation trials for each lemur). To minimize potential order effects and experimenter bias, the list of possible pairs was randomized and run in either forward or backward order, with order being counterbalanced across gender and species.

During each trial, a researcher placed two food items on the tray and handed it to a keeper. The keeper entered one of the night/holding enclosures and presented the tray to the designated lemur. A selection was determined when the focal lemur grasped and removed one of the two food items from the tray. Other lemurs rarely attempted to approach the keeper during a trial. If a lemur did attempt to interfere with a trial, the keeper would adjust their position so that only the designated lemur could select one of the food items. The lemur was given several seconds to make a choice. If the lemur did not make a choice within several seconds or moved away from the tray, the tray was re-presented to the lemur. If no choice was made after three presentation attempts, that trial was recorded as “no choice.” Up to 30 trials were run for each lemur in a day, and typically at least 5–10 trials were run consecutively for any one lemur at a time. Each trial took approximately 30 s to run (total of 20–30 trials each day per lemur; 2–4 lemurs tested each day).

Data were collected by recording both the food item selected and the position of that item for any given trial. The experimenter also recorded whether a lemur consumed the food item after selecting it. To minimize both experimenter and presenter bias, prior food selections were not discussed between the experimenter and presenter, and presentations of all food items were randomized for order and position. Each preference assessment took 2–3 days for each lemur, and the entire study took a total of 26 days.

Because of the small sample size for two of the four species included in the study, differences in food selections were determined by comparing the means and standard errors of the means for each species. Reported differences were based on non-overlapping standard errors of the means.

### Results and Discussion

**Figure 1:**
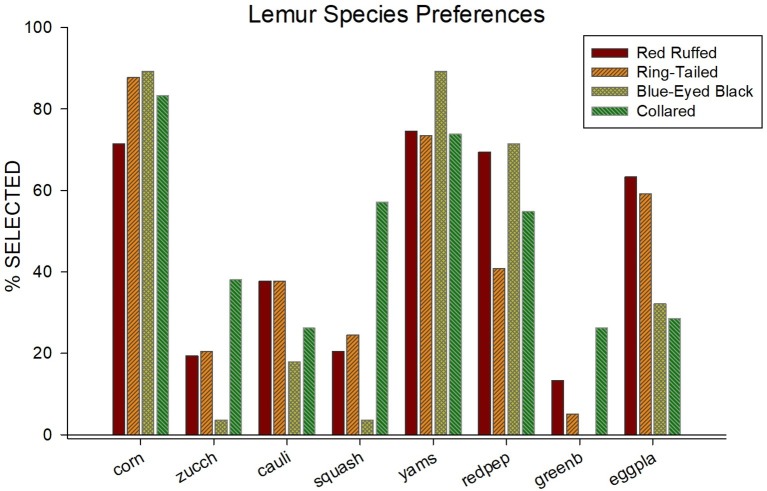
Percentage of selection (out of 14 times presented) for all eight of the food items used in the paired-choice preference assessment across all four species.

Figure 1 illustrates the food selections for each of the four species. Food items are listed across the x-axis, and percentage of times each item was selected are listed. Overall, a consistent pattern emerged across species for how often a food item was selected. Corn, yams, and red peppers were ranked, respectively, the first, second, and third most selected items overall. Green beans, squash, and zucchini were ranked, respectively, the least, second least, and third least selected items overall.

Table 1 provides the average and standard error for the number of times a food item was selected for each of the four species in the study. In addition to the obvious similarities in food selection among species shown in Figure 1, there are also differences in food selections. For instance, while corn was one of the most often selected food items for all four of the species, it was selected less often by the red ruffed lemurs (*M* = 10.00, SE = 0.44) when compared to the other three species (ring-tailed, *M* = 12.29, SE = 0.64; blue-eyed black, *M* = 12.50, SE = 1.50; collared, *M* = 11.67, SE = 0.88). Squash was selected in more than half of the trials for the collared lemurs (*M* = 8.00, SE = 1.00), but was selected in less than a third of the trials for the other three species (red ruffed, *M* = 2.86, SE = 1.18; ring-tailed, *M* = 3.43, SE = 0.90; blue-eyed black, *M* = 0.50, SE = 0.50). Yams were one of the most often selected food items for all four species (red ruffed, *M* = 10.43, SE = 0.65; ring-tailed, *M* = 10.29, SE = 0.18; blue-eyed black, *M* = 12.50, SE = 0.50; collared, *M* = 10.33, SE = 0.67). Green beans were one of the least selected items for the red ruffed, ring-tailed, and collared lemurs (red ruffed, *M* = 1.86, SE = 0.77; ring-tailed, *M* = 0.71, SE = 0.47; collared, *M* = 3.67, SE = 1.20), and were never selected by the blue-eyed black lemurs.

**Table 1:** Average number of times a food item was selected (out of a possible 14 presentations) by each of the four lemur species.

Food item	Red ruffed	Ring-tailed	Blue-eyed black	Collared
Corn				
Mean	10.00^B^	12.29	12.50	11.67
(SE)	(0.44)	(0.64)	(1.50)	(0.88)
Zucchini				
Mean	2.71	2.86	0.50^B^	5.33^A^
(SE)	(1.27)	(1.24)	(0.50)	(1.20)
Cauliflower				
Mean	5.29	5.29	2.50	3.67
(SE)	(1.06)	(0.52)	(2.50)	(1.33)
Squash				
Mean	2.86	3.43	0.50^B^	8.00^A^
(SE)	(1.18)	(0.90)	(0.50)	(1.00)
Yams				
Mean	10.43	10.29	12.50^A^	10.33
(SE)	(0.65)	(0.18)	(0.50)	(0.67)
Red pepper				
Mean	9.71^a,b^	5.71^b,c^	10.00^c^	7.67^a,c^
(SE)	(0.71)	(1.51)	(0.00)	(1.20)
Green bean				
Mean	1.86^a^	0.71^a^	0.00^B^	3.67^a^
(SE)	(0.77)	(0.47)	(0.00)	(1.20)
Eggplant				
Mean	8.86^a^	8.29^b^	4.50	4.00^a,b^
(SE)	(0.70)	(1.43)	(4.50)	(1.73)

*For remaining food selections, differences between species are marked with the same lower-case letters, either^a, b^ or^c^*.

A *Species that selected a food item more than all other species (based on non-overlapping standard errors of the means)*.

B *Species that selected a food item less than all other species*.

A similar pattern of selection was also present within each species. As evidence for similarities in selection, more than half of all standard errors of the mean food selections for each species were less than 1, and another third were less than 1.5. Two exceptions occurred, however, within the blue-eyed black lemurs: eggplant was selected nine times and cauliflower was selected five times by one lemur but never by the other.

It is worth noting that the position of the presented food items (on the right or left) appeared to have little effect on the food selections. For all 19 lemurs, items on the left were selected 47.95% of the time. The strongest position bias for any one lemur was a left item selection of 61.7%. Finally, when a food item was selected, it was almost always consumed (97.48%). Therefore, the preference for food items was based on their appeal as consumable food.

## Experiment 2: Enrichment Evaluation

Experiment 1 produced systematic paired rankings of food items that allowed them to be arranged in a preference order and used to select potential enrichment items. The purpose of Experiment 2 was to evaluate the relative effects of high- and low- preferred enrichment items, with a control (Baseline) condition in which the bamboo feeder was presented with no food items. Previous research demonstrated that food enrichment placed in hanging devices was effective in producing increased foraging and natural foraging postures in black and white ruffed lemurs (Britt, 1998). Based on this report, we hung bamboo dispensers in two trees in the lemurs’ enclosure during 1-h evaluation periods. The dispensers contained high-preferred food items, low-preferred food items, or no items. Evidence that food items increased interactions with the dispenser, enclosure use, and general activity was taken as evidence of enrichment, while differences between the effects of high- and low-preferred items provided evidence that the paired-choice assessments were useful in selecting enrichment items.

### Method

#### Subjects and Enclosures

Subjects included three of the seven ring-tailed lemurs, two new ring-tailed lemurs, and all three of the collared lemurs from Experiment 1 for a total of eight lemurs. Lemurs were chosen for Experiment 2 because they were the animals exhibited where the study was conducted. Due to limited direct access to the two new ring-tailed lemurs, no additional preference assessments were conducted. However, because there was low variability in the items selected across the ring-tailed lemurs during Experiment 1, this was not a major concern. In addition, for the first session, only four of the five ring-tailed lemurs were on exhibit. Five ring-tailed lemurs were on exhibit until session 14, when the fifth lemur was removed for the remainder of the study due to illness. Therefore, half of all the sessions (sessions 1 and 14–24) were run with four rather than five ring-tailed lemurs. All lemurs were observed in the outdoor island exhibit previously described in Experiment 1.

#### Materials

Materials included the eight food items assessed in Experiment 1. During experimental conditions, the food items were cut in 2–3 cm × 2–3 cm squares and placed in one of two 61 cm × 10 cm bamboo dispensers. Each bamboo dispenser had eight 4-cm diameter holes that allowed food to be withdrawn from it. Other materials included Palm® handhelds used to record behavioral data, an Event-PC program that was run on the Palm® handhelds and designed specifically for this experiment by Dr. James Ha at the University of Washington, and a notebook used to record potential errors and additional observations/field notes that occurred during a session.

#### Design and Procedure

**Figure 2:**
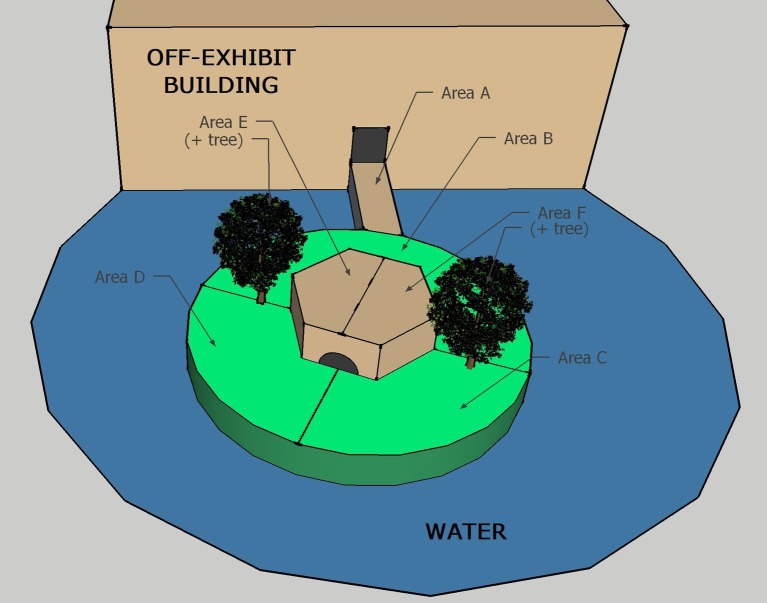
Diagram of the ring-tailed/collared lemur exhibit, as viewed from above. Capital letters represent each area, and lines represent their boundaries. The structure above Area A shows the holding/nighttime exhibit (lemurs reach the island exhibit by crossing Area A). The trees in Area E and Area F are where the devices were placed during all three conditions (BL, LP, HP).

A modified scan sampling procedure (Altmann, 1974) was used to record behaviors during all sessions. The exhibit was divided into six possible coding areas. Figure 2 shows the ring-tailed/collared lemur exhibit, with the coding areas labeled A through F. One of seven mutually exclusive and exhaustive behaviors listed in Table 2 was recorded for each animal present in that location during each sample. In other words, only one behavior was recorded for each animal in the coding area being sampled. A coding area was sampled each 30 s over 1 h for a total of 120 area-behavior samples.

**Table 2:** Behaviors and definitions for each response categorized in the ethogram.

Behavior	Definition
Active (A)	Movement around the enclosure, eating any edible items, or interacting with objects within the enclosure (other than the bamboo dispensers).
Dispenser-Directed (DD)	Manipulating one of the two bamboo dispensers used in the study. If a lemur is contacting a bamboo dispenser while eating, it is still recorded as DD.
Inactive (I)	Lying down or sitting in the enclosure. If lemur is contacting another lemur inactively (e.g., while lying down with no motion), this is still considered inactive.
Grooming (G)	Licking or manipulating own body (usually involving licking of body).
Interacting with Same Species (SS)	Orienting towards and/or actively contacting a lemur of the same species.
Interacting with Different Species (DS)	Orienting towards and/or actively contacting a lemur of a different species.
Other (O)	A behavior not listed above, or not being able to observe what a lemur is doing.

Because we were not able to observe all of the exhibit from any one area while distinguishing reliably between individuals within a species, for each area sample, we recorded instead the number of animals within a species that engaged in any of the coded behaviors in the sampled area. For example, at the start of the session, the observer(s) recorded the number of ring-tailed and collared lemurs engaged in any of the seven possible behaviors for Area A. Only behaviors occurring within Area A were recorded for that interval. During the following sampling interval, the same procedure was followed for Area B. The sample area was successively changed from A to F, and then the cycle was repeated beginning with Area A each time, creating a total of 20 samples per area for each session (3 min to cycle through the six areas).

A potential limitation of this technique is that individuals could be observed in more than one area during each scan. For instance, between 30-s observations, a lemur could move from Area A to Area B and thus be observed in both areas. To determine how often this may have happened, the total number of behaviors observed per species was counted for all 24 sessions. If a lemur was observed only once during each of the 3-min intervals required to observe all six areas, a total of 20 behaviors × the number of individuals in a particular species would be produced [60 observations for collared lemurs, 80 or 100 observations (depending on whether four or five individuals were on exhibit) for ring-tailed lemurs per session]. A number greater than this would suggest that one or more lemurs were counted more than once during a successive sample of the six areas, while a smaller number would suggest the animal moved so as not to be measured or was missed altogether. On average, both collared and ring-tailed lemurs were observed during 96% (SE = 1%) of all possible intervals recorded during all 24 sessions, suggesting that only a small number of possible observations per species were missed (2.4 observations for collared lemurs, 5.6 or 6.4 observations for ring-tailed lemurs per session). This result was due to either a lemur not being visible in an area or transitioning between areas during a sampling interval. Additional support for this assertion is evidenced by the fact that only two of the 24 sessions for both species produced a number greater than the total number of behaviors × the number of individuals observed.

Several minutes prior to each session, a keeper would place both bamboo dispensers in the exhibit. Each dispenser was tied approximately 1 m high to one of the two trees within the exhibit: one located in Area E and the other in Area F. There were three possible conditions during the experiment:

#### Baseline

During this condition, no food was placed in the bamboo dispensers. The empty bamboo dispensers were only placed on exhibit during the Baseline condition to avoid any potential habituation effects to the devices.

#### Low-Preferred Condition

Based on the preference assessment conducted in Experiment 1, the four least selected items (based on the combined means for the six ring-tailed and collared lemurs from Experiment 1 that were involved in Experiment 2) were determined. The four food items consisted of zucchini, cauliflower, red pepper, and green beans. During this condition, 50 g of each item was placed in each of the bamboo dispensers for a total of 200 g of food per dispenser. The two dispensers in this condition were placed on exhibit in the same manner as during the Baseline condition. It should be noted that while red pepper was the third most selected food item for all lemurs in Experiment 1, it was the fifth most selected item for the six lemurs in this experiment, hence why it was considered a low-preferred food item.

#### High-Preferred Condition

This condition was the same as LP, except using the four most selected items: corn, yams, eggplant, and squash. As per the Baseline and low-preferred (LP) conditions, the two dispensers were placed on exhibit as noted above.

A reversal design was used with each condition being returned to once. Following the second BL condition, the LP and HP conditions were run in reverse order to control for potential order effects (ABCACB reversal design). Eight sessions per condition were run—four sessions for each of the two times a condition was presented—for a total of 24 sessions. All sessions were run between 10:00 and 11:30 am on a Monday, Wednesday, or Friday. The entire study was conducted between June 25, 2004, and August 18, 2004.

Because of the small number of sessions (four) included in each of the two food-enrichment conditions, differences between the first and second time a condition was run were inspected visually. All differences showed changes of no greater than 30% between each time a condition was run, except for a 34% difference in same species interactions with the collared lemurs during the LP condition. This difference is discussed in the results and discussion section.

Interobserver agreement (IOA) was calculated based on total agreement (Poling et al., 1995) for 21% of all sessions conducted. All measures of total agreement were above 90%. However, because total agreement is calculated by determining the number of total observations for each observer, there is no guarantee that two observers were ever able to observe the same event during the same sampling interval. To estimate reliability without this possibility, we randomly sampled 20 observations from each of the five sessions where IOA was calculated and generated both percentage agreement and Kappa (percentage agreement corrected for chance agreement) for the 100 observations (Lehner, 1996).

The two independent observers agreed on all 100 ring-tailed lemur observations, which generated a Kappa value of 1. They agreed on 86.27% of the collared lemur observations, with a Kappa value of 0.68. Fleiss (1981) suggests that Kappa values > 0.6 are good, while values >0.75 are excellent.

SigmaStat 3.1® was used to run all the statistical analyses. The data for the behaviors and areas observed were split into 1/2-h bins (0–30 min, 31–60 min), and both 1/2-h bins and species were analyzed separately. Behaviors were split into 1/2-h bins because most of the food was removed within the first 1/2 h of introducing the food conditions, and therefore behavior after that point typically returned to Baseline levels of activity. All analyses reported passed normality and equal variance tests; therefore, we used a repeated-measures ANOVA with experimental condition as the blocking variable to examine the data. When significant differences (*p* < 0.05) were found, *post hoc* pairwise comparisons (Tukey test) were used to compare differences among the three experimental conditions.

To examine overall enclosure use, a measure of entropy (Shannon, 1948) was generated for each session. Entropy measures randomness across a set of variables and therefore produces a single measure of the total variability of enclosure use across the six possible areas. The measures of entropy were calculated by the formula

$$H=-\Sigma p\;\left(i\right)\;\log\;p\;\left(i\right)\text{,}$$

where *p*(*i*) is the proportion of time spent in *i*_th_ area. This formula produces a number from 0 to 1, with a higher value of *H* demonstrating more variability in overall enclosure use. Entropy was selected as a measure of variability in enclosure use [over a spread of participation index (SPI); Dickens, 1955; Hedeen, 1982; Plowman, 2003] because it is sensitive to small sets of variables and does not require a modified formula to accurately handle unequal enclosure zones. The same statistical analyses as listed above were then tested on the values of entropy.

### Results and Discussion

**Figure 3:**
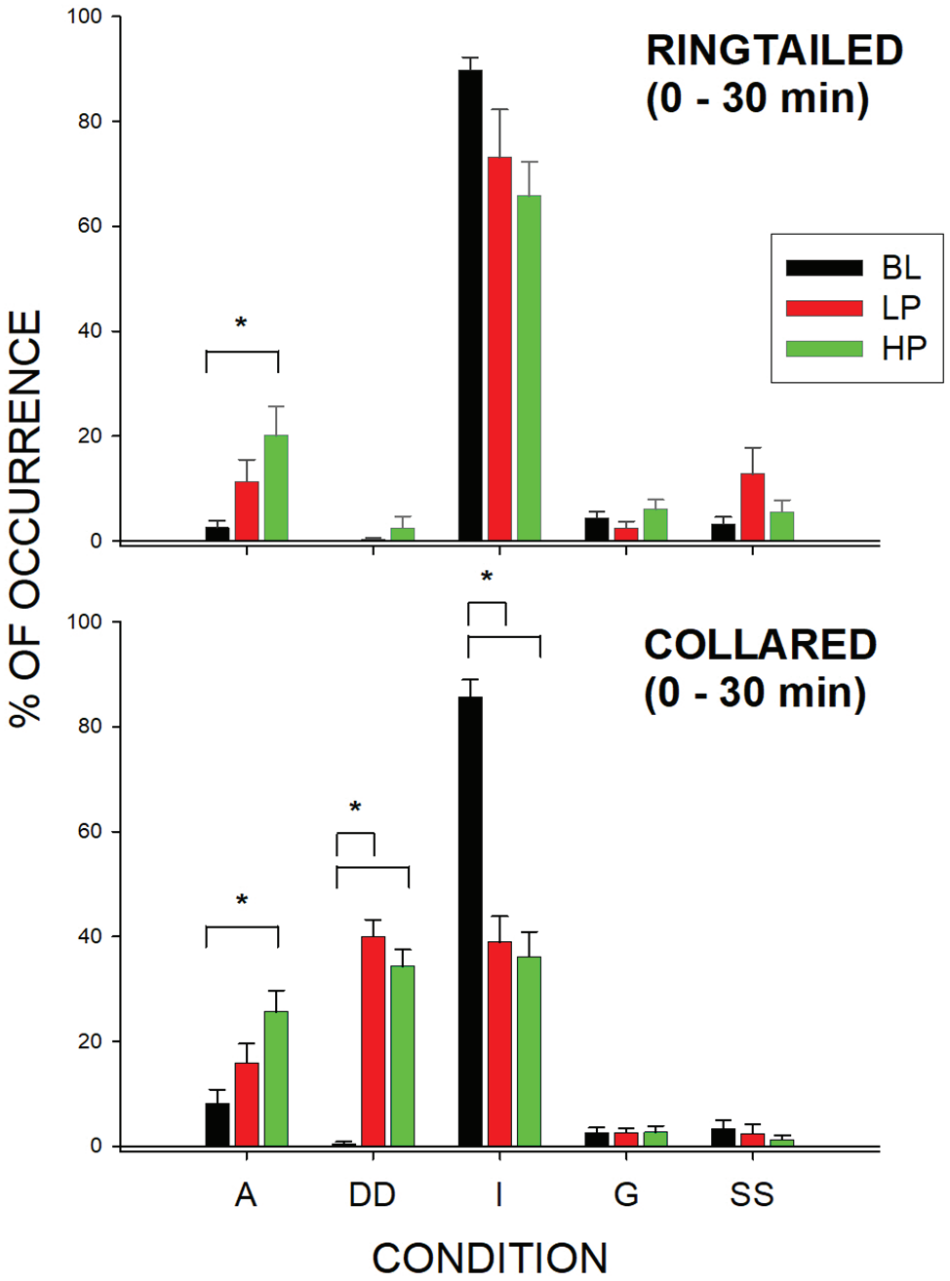
Mean percentage of occurrence (with SE bars) in the first 1/2 h for the Active (A), Dispenser-Directed (DD), Inactive (I), groom (G), and Same Species (SS) behaviors across all three conditions (BL, LP, and HP). The top graph shows the ring-tailed lemurs’ behaviors, and the bottom graph shows the collared lemurs’ behaviors. Asterisks and solids lines indicate significant differences between two conditions (*p* < 0.05).

Figure 3 shows the overall distribution of behaviors in the first 1/2 h for both species across all three conditions. Two of the behaviors that could be coded [Interacting with a Different Species (DS) and Other (O)] were never observed during the study, and therefore were not analyzed or graphed (see Table 2 for definitions of these responses).

#### Active and Inactive Behaviors

Ring-tailed lemurs showed a significant change in active behaviors (*F*_2,21_ = 5.30, *p* = 0.019), *d* = 0.65. Active (A) behaviors significantly increased during the HP condition compared to the BL condition (*p* = 0.015). There was also a significant change in Active behaviors for the collared lemurs (*F*_2, 21_ = 10.57, *p* = 0.002), *d* = 0.955. Active behaviors significantly increased during the HP condition compared to the BL condition (*p* = 0.001). For both species, the data therefore suggest that the greatest increase in Active behaviors was observed during the HP condition in the first 1/2 h.

The collared lemurs spent much of their time in the first 1/2 h of BL engaged in Inactive (I) behaviors. This changed significantly during the experiment (*F*_2, 21_ = 80.36, *p* < 0.001), *d* = 1.0, with Inactive behaviors decreasing during the LP condition (*p* < 0.001) and the HP condition (*p* < 0.001). Ring-tailed lemurs showed no significant changes in Inactive behaviors in the first 1/2 h. However, their Inactive behaviors decreased from 89.8% (SE = 2.4) to 73.2% (SE = 9.1) and 65.8% (SE = 6.5) for the LP ad HP conditions, respectively. For the collared lemurs, both food conditions had similar effects in reducing Inactive behaviors in the first 1/2 h compared to BL. For the ring-tailed lemurs, the data suggest that the HP condition had a greater effect than the LP condition in reducing Inactive behaviors during the first 1/2 h.

#### Dispenser-Directed Behaviors

The collared lemurs significantly increased their Dispenser-Directed (DD) behaviors in the first 1/2 h (*F*_2, 21_ = 71.14, *p* < 0.001), *d* = 1.0. During BL, the collared lemurs engaged in few Dispenser-Directed behaviors, although the frequency increased significantly during the LP condition (*p* < 0.001) and the HP condition (*p* < 0.001). Like the change in Inactive behaviors for the collared lemurs during the first 1/2 h, both food conditions increased Dispenser-Directed behaviors. Therefore, the presence of food within the dispensers produced the Dispenser-Directed behaviors, rather than the type of food present. The ring-tailed lemurs showed no significant changes in Dispenser-Directed behaviors for any of the behaviors during the first 1/2 h.

#### Second 1/2-h Effects

**Figure 4:**
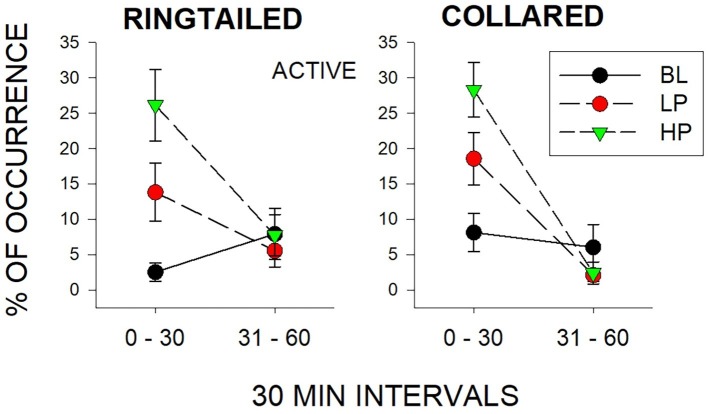
Mean percentage of occurrence (with SE bars) for the Active behaviors across all three conditions (BL, LP, and HP). The graph on the left is for the ring-tailed lemurs and the graph on the right is for the collared lemurs. The x-axis is split into 1/2-h intervals for the full hour of observation.

The only significant differences observed in the second 1/2 h were for Same-Species interactions (SS) with the collared lemurs (*F*_2, 21_ = 7.47, *p* = 0.006), *d* = 0.837. These Same-Species interactions were significantly higher during the LP condition when compared to both the BL condition (*p* = 0.009) and HP condition (*p* = 0.02). Therefore, the effects of both food conditions appeared to be short lived. Almost all changes in behaviors compared to Baseline were no longer observed by the second 1/2 h of observation. Figure 4 demonstrates this trend for the Active behaviors. As described previously, there were increases in both the LP and HP conditions when compared to BL for both species in the first 1/2 h. However, Active behaviors returned to Baseline levels of occurrence for both species during the LP and HP conditions in the second 1/2 h.

It was unclear why Same-Species interactions among the collared lemurs increased in the second 1/2 h, and more specifically, for the LP condition and not the HP condition. It was unlikely that the fewer desirable food items in the LP condition resulted in increased aggression, since the observers noted few instances of aggressive displays, and there was no demonstration of similar Different-Species interactions. The lower number of desirable items in the LP condition possibly increased later social foraging strategies, or there may have been an order effect. Most of the Same-Species interactions occurred during the second LP condition (first LP: *M* = 8.34, SE = 8.34; second LP: *M* = 42.26, SE = 10.37), when the LP condition followed the HP condition.

#### Overall Enclosure Use

**Figure 5:**
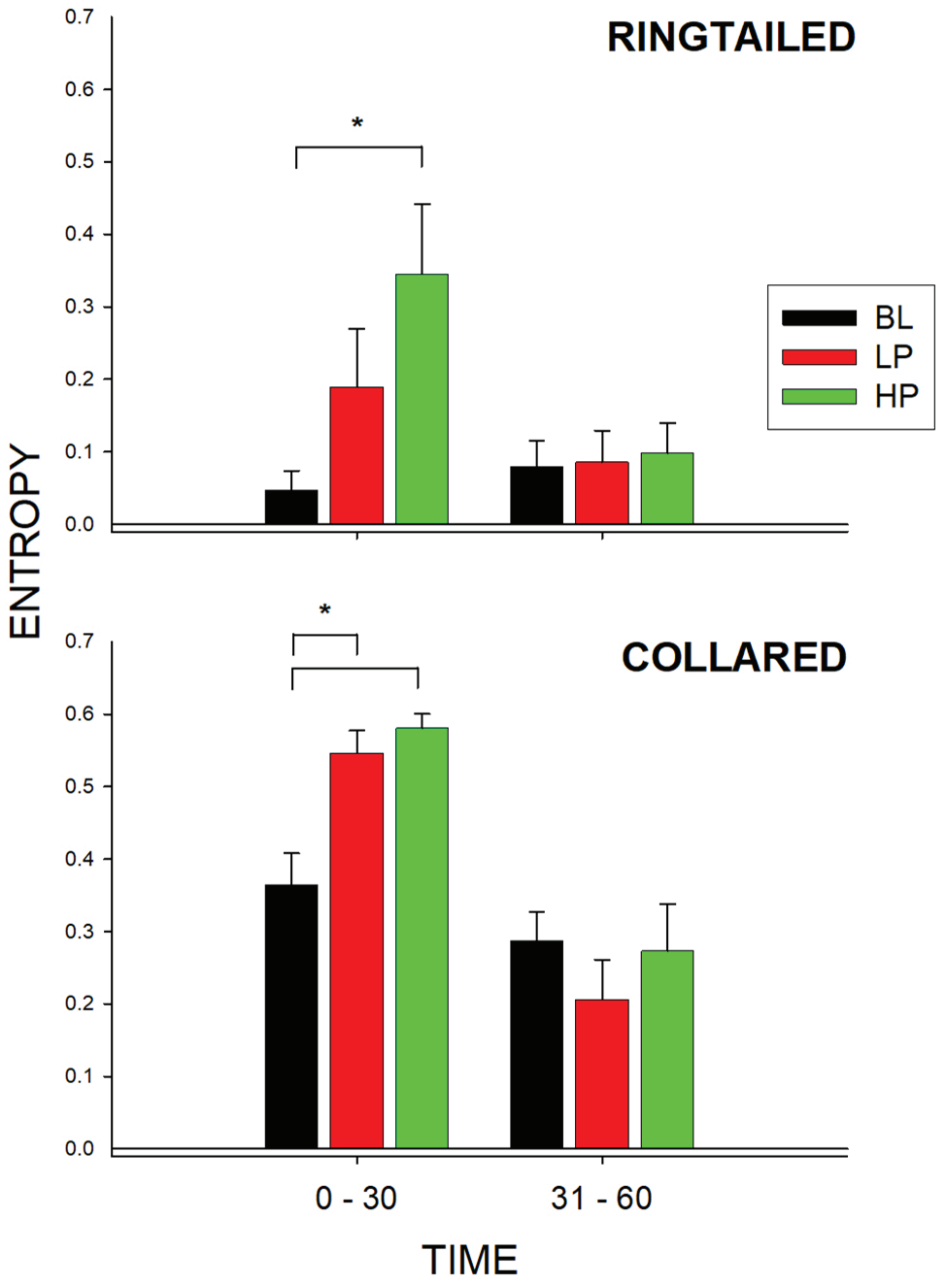
Mean entropy value (with SE bars) for the three conditions (BL, LP, and HP). The x-axis is split into 1/2-h intervals for the full hour of observation. The top graph shows the ring-tailed lemurs’ entropy values, and the bottom graph shows the collared lemurs’ entropy values. Asterisks and solids lines indicate significant differences between two conditions (*p* < 0.05).

Figure 5 shows the entropy values for both species and during both 1/2-h bins. As described previously, entropy was used to measure the total variability of enclosure use across the six possible areas within the lemur exhibit. A higher value of entropy indicates greater overall enclosure use for that species. There was a significant change in the entropy values for the collared lemurs (*F*_2, 21_ = 10.387, *p* = 0.002), *d* = 0.951. There was a significant increase in the entropy value from BL to both the LP condition (*p* = 0.008) and the HP condition (*p* = 0.002). For the ring-tailed lemurs, during the first 1/2 h, there was also a significant change in the entropy values (*F*_2,21_ = 4.109, *p* = 0.039), *d* = 0.498. There was a significant increase in the entropy value from the BL condition to the HP condition (*p* = 0.031). For the collared lemurs, both food conditions had similar effects on increasing overall enclosure use in the first 1/2 h compared to Baseline. For the ring-tailed lemurs, the data suggest that the HP condition had a greater effect than the LP condition in increasing overall enclosure use during the first 1/2 h of observation.

**Figure 6:**
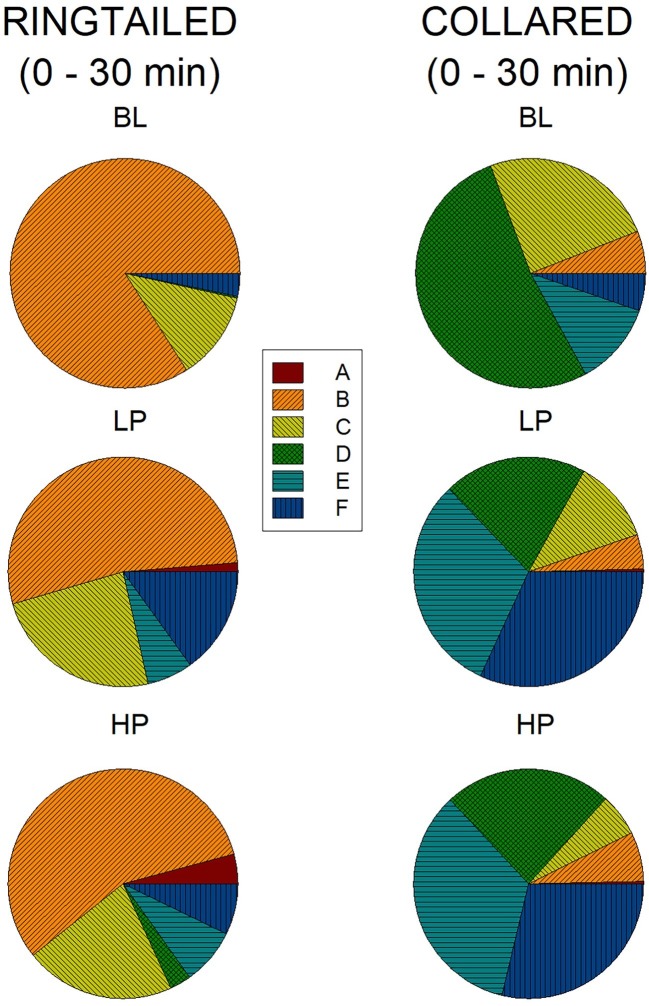
Mean percentage of area use in the first 1/2 h across the three conditions for both species. The graphs on the left are for the ring-tailed lemurs and the graphs on the right are for the collared lemurs. The top graphs are for the Baseline (BL) condition, the middle graphs for the low-preferred (LP) condition, and the bottom graphs for the high-preferred (HP) condition. Area E and F included the trees, where the devices were placed.

Figure 6 represents the percentage of area use for all six areas across all three conditions during the first 1/2 h. During Baseline, the ring-tailed lemurs spent 84% (SE = 12.18) of their time in Area B. This time decreased to 53.26% (SE = 16.97) and 56.53% (SE = 14.01) during LP and HP, respectively. Most other areas increased in use during the LP and HP conditions compared to Baseline. During the first 1/2 h of Baseline, the collared lemurs spent 24.71% (SE = 6.97) of their time in Area C, 52.27% (SE = 9.77) in Area D, 11.77% (SE = 7.58) in Area E, and 5.31% (SE = 2.41) in Area F. During both the LP and HP conditions, area use was more evenly distributed, with Area D decreasing (LP: *M* = 20.18%, SE = 4.69; HP: *M* = 23.67%, SE = 2.85), and Area E and F (which held the devices) increasing (Area E, LP: *M* = 30.97%, SE = 5.30; HP: *M* = 34.36%, SE = 5.22. Area F, LP: *M* = 31.89%, SE = 6.72; HP: *M* = 28.74%, SE = 2.93).

## General Discussion

Experiment 1 was successful in rapidly and systematically ranking the preferences of eight food items for all four species of lemur in the study, showing a high degree of similarity in food preferences within and between species. As noted previously, except for minor exceptions (e.g., blue-eyed black lemurs’ selection of cauliflower and eggplant), there were several similarities in the food selections both within and between a species. Because the food selections showed a clear and consistent ranking across all the lemurs, we were able to divide the choices into two categories: high- and low-preferred items. These categories facilitated testing the effects of food preferences on enrichment activities in Experiment 2.

In Experiment 2, presenting the high-preferred food items decreased Inactive behaviors and increased Active behaviors in the first 1/2 h, both with respect to BL. Presenting the low-preferred food showed similar trends with respect to the BL condition, but only the decrease in Inactive behaviors in the collared lemurs was significant. Although the average changes in behavior in the HP condition were consistently higher than those in the LP condition, there were no significant differences between the two experimental conditions.

Similarly, during the first 1/2 h following the presentation of food items, there was greater use of the enclosure (as measured by entropy values) for both species during one or both experimental conditions when compared to the Baseline. During Baseline, the ring-tailed lemurs spent almost all their time in Area B, while the collared lemurs spent more than half of their time in Area D. During both food conditions, times in Area B for the ring-tailed lemurs and Area D for the collared lemurs decreased, while there was an increase in most of the other areas within the enclosure. This change was due directly to the lemurs increasing the time they spent interacting with or remaining near the bamboo dispensers during the food conditions.

That food enrichment effects were largely confined to the first 1/2 h following the introduction of the bamboo dispensers indicates that the effect of our enrichment manipulation was limited to a relatively short time around the presentation of food. A larger amount of food (a total of 400 g of food was present in both dispensers) may have increased activity beyond the first 1/2 h. Since changes in the behaviors of the lemurs were directly related to the time it took the lemurs to consume the food, it seems worthwhile to investigate the effects of providing enrichment manipulations that require more extensive foraging activities. Distributing the bamboo dispensers more widely or making extraction of the food more difficult may have greater long-term effects.

It is worth noting that the presentation of the food may have interacted with the niche-related foraging repertoires of the two species. Ring-tailed lemurs are the most terrestrial of all lemur species in their habits and foraging activities (Duke University, 2005). Although the ring-tailed lemurs showed similar findings to the collared lemurs in terms of increased Active behaviors, decreased Inactive behaviors, and increased overall enclosure use, they rarely interacted with the suspended bamboo dispensers. Instead, during both high- and low-preferred experimental conditions, the ring-tailed lemurs remained below the hanging dispensers, picking up food that the collared lemurs dropped while manipulating the enrichment devices. This behavior suggests that for the ring-tailed lemurs, putting the bamboo holders on the ground might encourage more direct feeder interaction. Future research could be directed at comparing hanging vs. floor feeding enrichment for ring-tailed lemurs, as well as assessing preferences for both types of placement.

Ring-tailed lemurs are also known to shift their foraging patterns from fruit or leaves hanging on trees to fruit on the ground, depending on whether fruit and leaves have recently bloomed (Mertl-Millhollen et al., 2003). Therefore, it is possible that lemurs in captivity also change the percentage of time spent terrestrially based on the time of year. Future studies could examine differences in the effectiveness of hanging vs. non-hanging enrichment in ring-tailed lemurs during different seasons. Regardless, it is worth noting that one important component of naturalistic enrichment is that it interacts with species-typical behavioral repertoires, which is particularly true for foraging behavior. Environmental enrichment provides functionally related foraging opportunities for all species, which means that a better understanding of the natural history of any animal should facilitate the implementation of any enrichment practice.

### Enrichment Selection and Systematic Assessment

Our data support the use of paired-choice preference assessments for comparing multiple small, easily presented stimuli such as food items. Systematic comparisons of a limited number of alternatives produce empirically evaluated differences in a relatively short period of time, allowing multiple individuals to be assessed in a way that can apply to the preferences of groups. Preferences for available foods or other items could easily be run daily using a sample of the captive population and would help guide the type of enrichment to be used for that group. Preference assessments also can be used to determine differences between individuals in a group and thus help individualize the types of enrichment used. The main point is that preference assessments such as these can bypass the trial-and-error process of enrichment selection, and instead focus on using data to guide the selection of possible enrichment to better improve their effectiveness.

Finally, we examined environmental enrichment only as it applied to small manipulable food sources. As other researchers have noted, enrichment can also refer to physical and social stimuli and human-animal interactions (Mellen and MacPhee, 2001; Alligood and Leighty, 2015). It seems likely that these more abstract forms of enrichment could also be selected based on successive pairings of alternatives and inspection of choice behavior. Even with enrichment procedures not directly testable through paired choices, such as access to keepers or husbandry training procedures, stimuli selected during a preference assessment could be paired with these events and therefore make it possible to select and test most types of potential enrichment systematically.

## Data Availability

The datasets generated for this study are available on request to the corresponding author.

## Ethics Statement

The animals in this study were reviewed and approved by the IU – Bloomington IACUC and the Indianapolis Zoo Research Committee.

## Author Contributions

All authors listed have made a substantial, direct and intellectual contribution to the work, and approved it for publication.

### Conflict of Interest Statement

The authors declare that the research was conducted in the absence of any commercial or financial relationships that could be construed as a potential conflict of interest.
